# Comprehensive and Facile Synthesis of Some Functionalized Bis-Heterocyclic Compounds Containing a Thieno[2,3-*b*]thiophene Motif

**DOI:** 10.3390/ijms13022263

**Published:** 2012-02-20

**Authors:** Yahia N. Mabkhot, Assem Barakat, Abdullah M. Al-Majid, Saeed A. Alshahrani

**Affiliations:** Department of Chemistry, Faculty of Science, King Saud University, P.O. Box 2455, Riyadh 11451, Saudi Arabia; E-Mails: amajid@ksu.edu.sa (A.M.A.-M.); saddah096@hotmail.com (S.A.A.)

**Keywords:** pyrazole, nicotinic acid, thieno[2,3-*b*]thiophene, enaminone, heterocycle

## Abstract

A comprehensive and facile method for the synthesis of new functionalized bis-heterocyclic compounds containing a thieno[2,3-*b*]thiophene motif is described. The hitherto unknown bis-pyrazolothieno[2,3-*b*]thiophene derivatives **2a**–**c**, bis-pyridazin othieno[2,3-*b*]thiophene derivatives **4**, bis-pyridinothieno[2,3-*b*]thiophene derivatives **6a**,**b**, and to an analogous bis-pyridinothieno[2,3-*b*]thiophene nitrile derivatives **7** are obtained. Additionally, the novel bis-pyradazinonothieno[2,3-*b*]thiophene derivatives **9**, and nicotinic acid derivatives **10**, **11** are obtained via bis-dienamide **8**. The structures of all newly synthesized compounds have been elucidated by ^1^H, ^13^C NMR, GCMS, and IR spectrometry. These compounds represent a new class of sulfur and Nitrogen containing heterocycles that should also be of interest as new materials.

## 1. Introduction

Heterocycles are well known for displaying a wide range of biological properties [1]. The structural diversity and biological importance of *N*-containing heterocycles have made them attractive synthetic targets over many years. Pyrazole and pyrazoline derivatives are in general well-known five-membered *N*-containing heterocyclic compounds [2,3]. Compounds with pyrazole ring are of interest due to their broad spectrum of biological activities as NOS inhibitors [4], monoamine oxidase inhibitor [5], antibacterial [6], and antiamoebic compounds [7]. Moreover, *N*-phenylpyrazole derivatives play an important role in antitumor screening [8] as well as potent antimicrobial products [9,10]. Furthermore, a number of pyridine and pyridazine derivatives are gaining synthetic interest in recent years due to their broad spectrum of biological activities [11]. On the other hand, many thiophene-containing compounds, including annulated compounds, exhibit biological activities [12,13]. Thienothiophene derivatives have been developed for different purposes in the pharmaceutical field and have been tested as potential antitumor, antiviral and antibiotic, antiglaucoma drugs, or as inhibitors of platelet aggregation [14–18]. In addition, thienothiophenes have potential applications in a wide variety of optical and electronic systems [19–21]. Furthermore, bis-heterocyclic have received great attention as being model compounds for main chain polymers [22–26]. It is also reported that many biologically active natural and synthetic products have molecular symmetry [27]. Keeping the above facts in mind and in continuation of our interest in the synthesis of bis(hetrocycles) [28–31], we describe herein a simple and efficient route for the synthesis of novel bis(enaminones) and studied their synthetic utilities as key intermediates for the synthesis of novel bis-heterocycles pyrazoles, pryridines, and pyridazine derivatives.

## 2. Results and Discussion

**Scheme 1:**
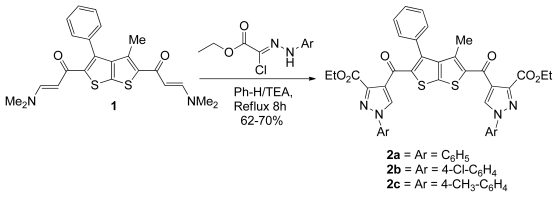
The synthesis of bis-pyrazole derivatives **2a**–**c**.

Recently, enaminones **1** were prepared by different synthetic approaches and their use as building blocks for the synthesis of a wide variety of heterocycles has been investigated [3,32–35]. The new bis(enaminones) **1** being now available to us prompted us to study its synthetic utility as key intermediate for novel bis(5- and 6-membered) heterocycles with the thieno[2,3-*b*]thiophene core. Thus, heating the bis(enaminone) with ethyl-2-(2-arylhydrazono)-2-chloroacetate which is commercially available in dry benzene and TEA (Tri ethyl amine) resulted in the formation of the bis(1-Phenyl-1-*H*-pyrazole-3-carboxylate) derivatives (**2a**) in 62% yield as depicted in Scheme 1.

The structure of compound **2a** was determined from its elemental and spectroscopic analyses. Its IR spectrum showed the presence of an absorption band characteristic for C=O as well as the presence of C=N absorption at 1627 cm^−1^. The ^1^H NMR spectrum of **2a** showed a singlet at 10.6 ppm due to the pyrazole ring (5-CH). It is noteworthy to mention that the bis(pyrazoles) **2a**, **2b** could also be prepared from **1** in 67 and 70% yields, respectively, using the above synthetic methodology (Scheme 1). The formation of the bis(pyrazole) **2a** from **2b** or **2c** is supposed to proceed through the formation of the nonisolable intermediates followed by the elimination of two molecules of the secondary amines.

**Scheme 2:**
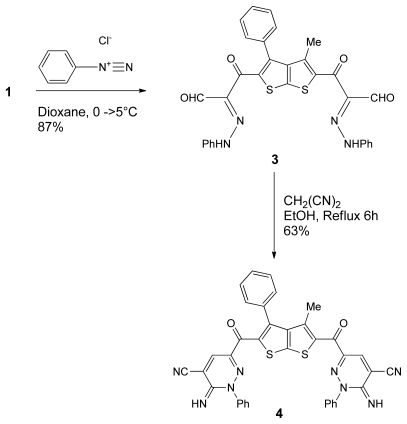
The synthesis of bis-pyridazine derivatives **4**.

Our study is now extended to include the synthesis of new bis(pyridines) and bis(pyridazines) derivatives. Thus, **1** was allowed to react with benzenediazoniumchloride which was prepared *in situ* from aniline in a HCl with aqueous NaNO_2_ in dioxane at 0–5°C to give a single product as examined by TLC. Elemental analyses and mass spectrum of the isolated product were completely in agreement with the molecular formula C_31_H_22_N_4_O_4_S_2_. The structure of the product, obtained in 87% yield, is assumed to be **3** according to the rationale outlined in Scheme 2. The later compound reacts with malononitrile in refluxing EtOH which undergoes intramolecular cyclization to give compound **4** in 63% yield. The ^1^H NMR spectrum of compound **4** was free of aldehydic proton and exhibited one characteristic singlet at 7.89 pmm integrated for 1H (for C–H of the pyridazine moiety). In addition, one singlet at 8.16 pmm integrated for 1H (for the NH-Pyridazine moietiy). Furthermore, the appearance of CN, and NH absorption at 2187, 3305 cm^−1^, respectively in the IR spectrum as well as its appearance as a broad singlet (8.16 pmm) in the ^1^H NMR spectrum strongly supported this assignment.

**Scheme 3:**
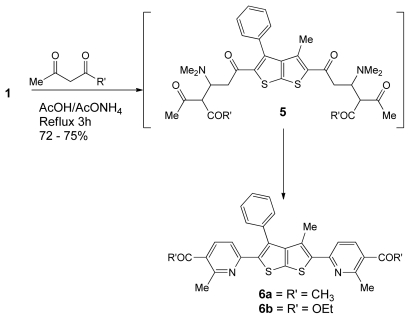
The synthesis of bis-pyridine derivatives **6a**,**b**.

Next, we have also described the synthesis of the new bis(pyridine) derivatives **6a**,**b** in 72–75% by the reaction of **1** with the corresponding acetone derivatives. Thus, reaction of **1** with acetylacetone in refluxing AcOH in the presence of NH_4_OAc afforded the corresponding bis(pyridine) derivative **6a** in 72% yields. Similarly, reaction of **1** with ethyl acetoacetate under the same reaction conditions gave the target bis(pyridine) **6b** in 75% yields as outlined in Scheme 3. It is assumed that enolates are initially added via 1,4-addition to the enaminones **1** to give the nonisolable intermediate **5**. Subsequent intermolecular cyclization via dimethylamine elimination gave the target compounds **6a**,**b**. The structures of compounds **6a**,**b** were inferred from different spectroscopic and analytical data.

**Scheme 4:**
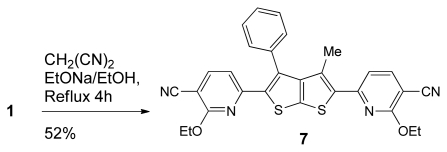
Synthesis of bis-pyridine nitrile derivatives **7**.

It is noteworthy to mention here that the bis-(heterocycle) **7** was also prepared from the corresponding enaminones **1** but in moderate yield, as depicted in Scheme 4.

**Scheme 5:**
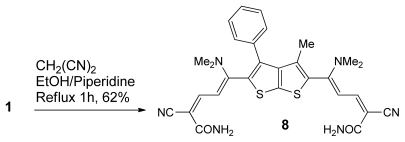
Synthesis of bis-dienamide **8**.

**Scheme 6:**
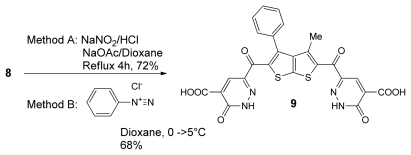
Synthesis of bis-(pyradazinone) derivatives **9**.

**Scheme 7:**
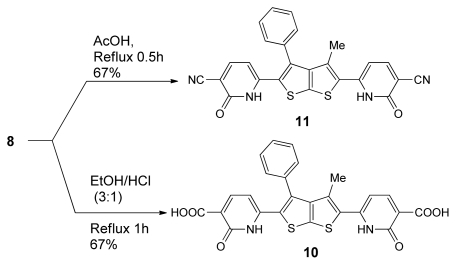
The synthesis of nicotinic acid derivatives **10**, **11**.

When enaminone **1** was treated with malononitrile in ethanolic piperidine under reflux for 1h which was a red crystalline product, bis-dienamide derivatives **8** was obtained in 62% yield (Scheme 5), used as key intermediate for the synthesis of a wide variety of bis-(pyridazinones) **9**, and bis-(pyridinones) **10**, **11** as drawn in Schemes 6 and 7.

**Figure 1:**
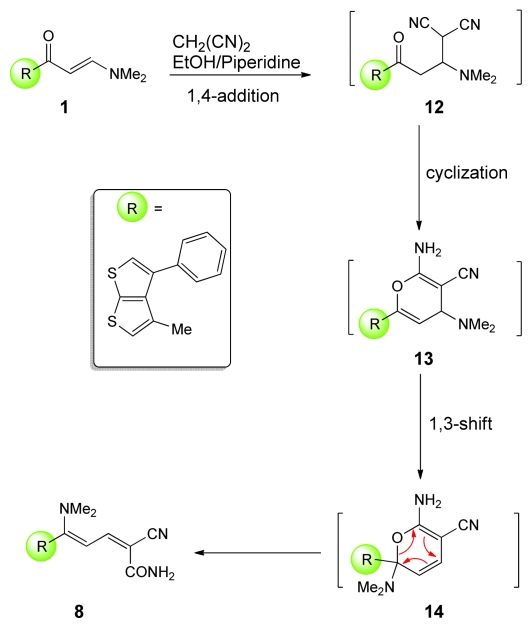
Proposed mechanism for the formation of bis-dienamide **8**.

In consideration of the reported data we believe that **8** was formed [36] via initial 1,4-addition of malononitrile across the C=C bond to yield **12** that cyclized to **13** and then rearranged to **14** via an allowed 1,3-nitrogen shift (Figure 1).

It is noteworthy to mention that the bis(pyradazinone) derivatives **9** could also be prepared from the appropriate bis-dienamide **8** [36]. Two methods were studied for the synthesis of **9**. In the first method, treating **8** with NaNO_2_/HCl in refluxing dioxane in the presence of NaOAc for 4 h afforded the final product in 72% yield. Alternatively, by coupling of **8** with phenyl diazonium salt gave the authentic product in 68% yield as drawn in Scheme 6.

**Figure 2:**
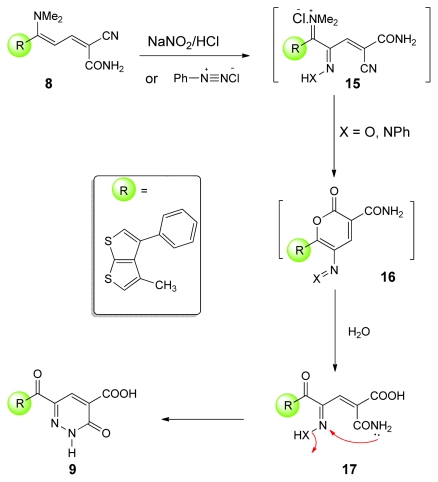
Proposed mechanism for the nitrozation and coupling reactions.

Consequently, a plausible mechanism for the formation of compounds **9** is illustrated in Figure 2. It is assumed that the initially formed **15** is subject to a cyclization to **16**, which is further hydrolysed to **17** under the reaction conditions. Finally, the lone pairs on the amide N-atom react with the oxime N-atom kicking out H_2_O producing **9.**

Conversions of **8** into nicotinic acid derivatives **10** were achieved by boiling in EtOH/HCl. When, however, **8** was heated under reflux in AcOH, nicotinic nitrile derivative **11** was obtained. The novel bis pyridinones **10**, **11** were assumed to be formed via nucleophilic attack of NH at the activated C=C bond of the enamine derivative to eliminate two molecules of Me_2_NH affording the final product as depicted in Scheme 7. The structures of compounds **10**, **11** were inferred from their spectroscopic and analytical data.

## 3. Experimental Section

M.P. were measured on a Gallenkamp melting point apparatus in open glass capillaries and are uncorrected. IR spectra were measured as KBr pellets on a Perking Elmer FT 1000 spectrophotometer. The NMR spectra were recorded on a Varian Mercury Jeol-400 NMR spectrometer. ^1^H-NMR (400 MHz) and ^13^C-NMR (100 MHz) were run in (DMSO-*d*_6_). Chemical shifts (δ) are referred in ppm and coupling constants *J* are given in Hz. Abbreviations for multiplicity are as follows: s (singulet), d (doublet), t (triplet), q (quadruplet), m (multiplet). Mass spectra were recorded on a Shimadzu GCMS-QP 1000 EX mass spectrometer at 70 eV. Elemental analysis was carried out on an Elementar Vario EL analyzer.

General procedure for the synthesis of compounds **2a**–**c** (GP1). To a soln of **1** (0.5 mmol, 0.21 g) in dry benzene (20 mL), ethyl 2-(2-arylhydrazono)-2-chloroacetate derivative (2 equiv., 1 mmol) was added in the presence of few drops of Et_3_N, thus the reaction mixture was heated under reflux for 8 h. The solid product was collected by filtration, and recrystallized from EtOH.

### Diethyl-4,4′-(3-Methyl-4-phenylthieno[2,3-b]thiophene-2,5-dicarbonyl)bis(1-phenyl-1H-pyrazole-3-carboxylate) (**2a**)

**2a** was prepared from ethyl 2-(2-phenylhydrazono)-2-chloroacetate following GP1, pale brown crystals, yield (62%); m.p. 206–208 °C; IR (ν_max_): 1716–1732 (C=O), 1627 (C=N) cm^−1;^ ^1^H-NMR (400 MHz, DMSO-*d*_6_): 1.30 (t, 3H, *J* = 6.9 Hz, CH_3_, ester), 1.96 (s, 3H, CH_3_), 4.25 (q, 2H, *J* = 16.6 Hz, CH_2_, ester), 7.36–7.76 (m, 15H, Ar-H), 10.6 (s, 1H, pyrazolo-H); ^13^C-NMR (100 MHz, DMSO-*d*_6_) δ (ppm): 14.49, 21.70, 66.25, 168.27, 192.12; MS *m/z* (%): 714 [M^+^, 1%], 240 (74), 166 (100), 98 (72); Anal. Calcd for C_39_H_30_N_4_O_6_S_2_: C, 65.53; H, 4.23; N, 7.84; S, 8.97; Found: C, 65.57; H, 4.21; N, 7.82; S, 9.

### Diethyl-4,4′-(3-methyl-4-phenylthieno[2,3-b]thiophene-2,5-dicarbonyl)bis(1-(4-chlorophenyl)-1H-pyrazole-3-carboxylate) (**2b**)

**2b** was prepared from Ethyl 2-(2-(4-chlorophenyl)hydrazono)-2-chloroacetate following GP1 as a yellow crystal, yield (67%); m.p. 236–238 °C; IR (ν_max_): 1710 (C=O), 1624 (C=N) cm^−1;^ ^1^H-NMR (400 MHz, DMSO-*d*_6_) δ (ppm): 1.30 (t, 3H, *J* = 6.9 Hz, CH_3_, ester), 1.96 (s, 3H, CH_3_), 4.25 (q, 2H, *J* = 16.6 Hz, CH_2_, ester), 7.36–7.96 (m, 13H, Ar-H), 10.6 (s, 1H, pyrazolo-H); ^13^C-NMR (100 MHz, DMSO-*d*_6_): 14.49, 21.70, 66.25, 168.27, 192.12; MS *m/z* (%): 782 [M^+^, 1.5%]; Anal. Calcd for C_39_H_28_Cl_2_N_4_O_6_S_2_: C, 59.77; H, 3.60; Cl, 9.05; N, 7.15; O, 12.25; S, 8.18; Found: C, 59.80; H, 3.58; Cl, 9.07; N, 7.14; S, 8.18.

### Diethyl-4,4′-(3-methyl-4-phenylthieno[2,3-b]thiophene-2,5-dicarbonyl)bis(1-(4-methoxyphenyl)-1H-pyrazole- 3-carboxylate) (**2c**)

**2c** was prepared from Ethyl 2-(2-(4-methylphenyl) hydrazono)-2-chloroacetate following GP1 as a yellow crystal, yield (70%); m.p. 240–242 °C; IR (ν_max_): 1728 (C=O), 1624 (C=N) cm^−1;^ ^1^H-NMR (400 MHz, DMSO-*d*_6_) δ (ppm): 1.2 (t, 3H, *J* = 6.9 Hz, CH_3_, ester), 1.96 (s, 3H, CH_3_), 2.22 (s, 3H, CH_3_), 4.25 (q, 2H, *J* = 16.6 Hz, CH_2_, ester), 7.13–7.66 (m, 13H, Ar-H), 10.5 (s, 1H, pyrazolo-H); ^13^C-NMR (100 MHz, DMSO-*d*_6_) δ (ppm): 8.2, 14.49, 21.70, 66.25, 168.27, 192.12; MS *m/z* (%): 742 [M^+^, 1.8%]; Anal. Calcd for C_41_H_34_N_4_O_6_S_2_: C, 66.29; H, 4.61; N, 7.54; O, 12.92; S, 8.63; Found: C, 66.32; H, 4.60; N, 7.52; S, 8.58.

### 3,3′-(3-Methyl-4-phenylthieno[2,3-b]thiophene-2,5-diyl)bis(3-oxo-2-(2-phenylhydrazono)propanal) (**3**)

To a stirred soln. of **1** (0.5 mmol, 0.21 g) in dioxane (15 mL) was cooled in an ice bath at 0–5 °C, a soln. of the benzenediazonium chloride [Freshly prepared by diazotizing aniline (1 mmol) in HCl (0.28 mL) with NaNO_2_ (2 mmol) in H_2_O (4 mL)] was added drop wise over a period of 20 min. The reaction mixture was kept in a refrigerator overnight. The solid product was collected by filtration, and recrystallized from EtOH to give yellow crystal, yield (87%); m.p. 176–178 °C; IR (ν_max_): 1591 (C=N), 1627–1639 (C=O), 3435 (NH) cm^−1;^ ^1^H-NMR (400 MHz, DMSO-*d*_6_) δ (ppm): 2.08 (s, 3H, CH_3_), 7.19–7.61 (m, 15H, Ar-H), 7.69 (s, 1H, N-H), 8.66 (s, 1H, CHO); ^13^C-NMR (100 MHz, DMSO-*d*_6_) δ (ppm): 14.13, 122.46, 128.62, 129.35, 129.96, 130.36, 130.54, 136.37, 145.83, 147.23, 187.13, 188.86. MS *m/z* (%):578 [M^+^, 45%]; Anal. Calcd for C_31_H_22_N_4_O_4_S_2_: C, 64.34; H, 3.83; N, 9.68; O, 11.06; S, 11.08; Found: C, 64.37; H, 3.80; N, 9.63; S, 11.14.

### 6-(5-(5-Cyano-6-imino-1,6-dihydropyridazine-3-carbonyl)-3-methyl-4-phenylthieno [2,3-b]thiophene-2-carbonyl)-3-imino-2,3-dihydropyridazine-4-carbonitrile (**4**)

To a stirred solution of 1 (0.5 mmol, 0.21 g) in EtOH (10 mL). Malononitrile (1 mmol, 0.07 g) was added and the resulting reaction mixture was heated under reflux for 6 h. The solid product was collected by filtration, and recrystallized from (EtOH). B was obtained as yellow crystal, yield (63%); m.p. > 320–322 °C; IR (ν_max_): 1593 (C=N), 1627 (C=O), 2187 (CN),3305 (NH) cm^−1;^ ^1^H-NMR (400 MHz, DMSO-*d*_6_) δ (ppm): 2.01 (s, 3H, CH_3_), 7.23–7.63 (m, 15H, Ar-H), 7.89 (s, 1H, CH, pyridazyl), 8.16 (s, 1H, N-H); ^13^C-NMR (100 MHz, DMSO-*d*_6_) δ (ppm): 14.13, 107.98, 115.98, 122.42, 128.42, 129.45, 129.96, 130.36, 130.54, 136.37, 145.83, 147.23, 154.22, 187.33; MS *m/z* (%): 674 [M^+^, 45%]; Anal. Calcd for C_37_H_22_N_8_O_2_S_2_: C, 65.86; H, 3.29; N, 16.61; O, 4.74; S, 9.50; Found: C, 65.84; H, 3.29; N, 16.63; S, 9.50.

General procedure for the synthesis of compounds **6a**–**c** (GP2). To a solution of 1 (0.5 mmol, 0.21 g) in acetic acid glacial (10 mL). Acetyl acetone derivatives (2 equiv., 1 mmol) was added in the presence of catalytic amount of ammonium acetate. The reaction mixture was heated under reflux for 3 h. The solid product was collected by filtration, and recrystallized from (EtOH).

### 1,1′-(6,6′-(3-methyl-4-phenylthieno[2,3-b]thiophene-2,5-diyl)bis(2-methylpyridine-6,3-diyl))diethanone (**6a**)

**6a** was prepared from acetylacetone following GP2 as a dark yellow crystal, yield (72%); m.p. 241–243 °C; IR (ν_max_): 1682 (C=O), 1624 (C=N) cm^−1;^ ^1^H-NMR (400 MHz, DMSO-*d*_6_) δ (ppm): 1.96 (s, 3H, CH_3_), 2.51 (s, 3H, COCH_3_), 2.62 (s, 3H, CH_3_, pyridyl), 7.29–7.59 (m, 5H, C_6_H_5_), 7.95 (d, 1H, *J* = 6.6 Hz, CH, pyridyl), 8.25 (d, 1H, *J* = 8.0 Hz, CH, pyridyl); ^13^C-NMR (100 MHz, DMSO-*d*_6_) δ (ppm): 14.49, 29.37, 30.55, 129.23, 129.55, 129.87, 134.79, 138.82, 141.84, 147.68, 151.23, 166.13, 194.1; MS *m/z* (%): 496 [M^+^, 3%]; Anal. Calcd for C_29_H_24_N_2_O_2_S_2_: C, 70.13; H, 4.87; N, 5.64; S, 12.91; Found: C, 70.18; H, 4.84; N, 5.6; 1; S, 12.96.

### Diethyl-6,6′-(3-methyl-4-phenylthieno[2,3-b]thiophene-2,5-diyl)bis(2-methylnicotinate) (**6b**)

**6b** was prepared from ethylacetoacetate following GP2 as a yellow crystal, yield (75%); m.p. 228–230 °C; IR (ν_max_): 1714 (C=O), 1622 (C=N) cm^−1;^ ^1^H-NMR (400 MHz, DMSO-*d*_6_) δ (ppm): 1.29 (t, 3H, *J* = 8.0Hz, CH_3_, ester), 1.98 (s, 3H, CH_3_), 2.5 (s, 3H, CH_3_, pyridyl), 4.25 (q, 3H, *J* = 8.0 Hz, CH_3_, ester), 7.14–7.58 (m, 5H, C_6_H_5_), 7.85 (d, 1H, *J* = 6.5 Hz, CH, pyridyl), 8.20 (d, 1H, *J* = 7.5 Hz, CH, pyridyl); ^13^C-NMR (100 MHz, DMSO-*d*_6_) δ (ppm): 14.49, 29.37, 30.55, 62.17, 129.23, 129.55, 129.87, 134.79, 138.82, 141.84, 147.68, 151.23, 166.13, 194.1; MS *m/z* (%): 556 [M^+^, 38%], 435 (12), 363 (48), 250 (55), 98 (100), 97 (45); Anal. Calcd for C_31_H_28_N_2_O_4_S_2_: C, 66.88; H, 5.07; N, 5.03; S, 11.52; Found: C, 66.91; H, 5.04; N, 5.02; S, 11.52.

### 6,6′-(3-methyl-4-phenylthieno[2,3-b]thiophene-2,5-diyl)bis(2-ethoxynicotinonitrile) (**7**)

To a solution of 1 (0.5 mmol, 0.21 g) in EtONa (Na 0.02g/EtOH 10 mL), malononitrile (1 mmol, 0.07 g) was added and the resulting reaction mixture was heated under reflux for 4 h. The solid product was collected by filtration, 7 was obtained as red crystal, yield (52%); m.p. > 330–332 °C; IR (ν_max_) : 1573 (C=N), 2200 (CN) cm^−1;^ ^1^H-NMR (400 MHz, DMSO-*d*_6_) δ (ppm): 1.32 (t, 3H, *J* = 8.0 Hz, CH_3_), 1.89 (s, 3H, CH_3_), 5.15–5.90 (q, 2H, *J* = 8.0 Hz, CH_2_), 7.22 (d, 1H, *J* = 7.5 Hz, CH), 7.36–7.43 (m, 5H, Ar-H), 8.44 (d, 1H, *J* = 7.5 Hz, CH). ^13^C-NMR (100 MHz, DMSO-*d*_6_) δ (ppm): 14.8, 29.58, 69.92, 103, 115, 119, 128.68, 128.81, 129.98, 133.61, 143,8, 149.1, 176, 180; MS *m/z* (%): 522 [M^+^, 12%], 492(6), 403(14), 149(100), 91(64); Anal. Calcd for C_29_H_22_N_4_O_2_S_2_: C, 66.64; H, 4.24; N, 10.72; S, 12.27; Found: C, 66.67; H, 4.23; N, 10.71; S, 12.25.

### 5,5′-(3-methyl-4-phenylthieno[2,3-b]thiophene-2,5-diyl)bis(2-cyano-5-(dimethylamino)penta-2,4- dienamide) (**8**)

To a solution of 1 (0.5 mmol, 0.21 g) in EtOH (10 mL), malononitrile (1 mmol, 0.07 g) was added in the presence of catalytic amount of piperidine. The reaction mixture was heated under reflux for 1 h. The solid product was collected by filtration, 8 was obtained as red crystal, yield (62%); m.p. 201–203 °C; IR (ν_max_): 1616 (C=O), 2193 (CN), 3388 (NH2) cm^−1;^ ^1^H-NMR (400 MHz, DMSO-*d*_6_) δ (ppm): 1.95 (s, 3H, CH_3_), 2.99–3.00 (s, 12H, N-CH_3_), 5.9(d, 1H, *J* = 12.8 Hz, CH), 7.2 (d, 1H, *J* = 12.8 Hz, CH), 7.32–7.90 (m, 5H, Ar-H), 8.2 (s, 2H, NH_2_); ^13^C-NMR (100 MHz, DMSO-d_6_) δ (ppm): 180.04, 176.68, 160.58, 149.18, 148.6, 133.6, 129.98, 128.80, 128.68, 119.20, 103, 79.12, 43.2, 14.85; MS *m/z* (%): 556 [M^+^, 1.2], 191 (6.1), 149 (100),73 (34); Anal. Calcd for C_29_H_28_N_6_O_2_S_2_: C, 62.57; H, 5.07; N, 15.10; S, 11.52; Found: C, 62.60; H, 5.04; N, 15.08; S, 11.48.

### 6-(5-(5-cyano-6-oxo-1,6-dihydropyridin-2-yl)-3-methyl-4-phenylthieno[2,3-b]thiophen-2-yl)-2-oxo-1,2-dihydropyridine-3-carboxylic acid (**11**)

To a solution of 8 (0.5 mmol, 0.28 g) in a mixture of EtOH/HCl (3:1, 10 mL) was refluxed for 1 h. The solid product was collected by filtration, and recrystallized from (EtOH). 11 was obtained as deep red crystal, yield (67%); m.p. 287–289 °C; IR (ν_max_): 1722–1631 (C=O), 3174 (OH), 3450 (NH) cm^−1;^ ^1^H-NMR (400 MHz, DMSO-*d*_6_) δ (ppm): 1.95 (s, 3H, CH_3_), 5.86 (d, 1H, *J* = 7.5 Hz, CH), 7.02 (s, 1H, NH), 7.14–7.90 (m, 5H, Ar-H), 8.34 (d, 1H, *J* = 7.5 Hz, CH), 11.35 (s, 1H, OH); ^13^C-NMR (100 MHz, DMSO-*d*_6_) δ (ppm): 14.44, 105.89, 123.71, 128.57, 129.87, 151.36, 162.88, 175.10; MS *m/z* (%): 485 [M^+^, 45%]; Anal. Calcd for C_25_H_15_N_3_O_4_S_2_: C, 61.84; H, 3.11; N, 8.65; S, 13.21; Found: C, 61.85; H, 3.10; N, 8.67; S, 13.24.

### 6-(5-(5-cyano-6-oxo-1,6-dihydropyridin-2-yl)-3-methyl-4-phenylthieno[2,3-b]thiophen-2-yl)-2-oxo-1,2-dihydropyridine-3-carbonitrile (**10**)

To a solution of **8** (0.5 mmol, 0.28 g) in acetic acid glacial (10mL), and the resulting mixture was refluxed for 30 min. The solid product was collected by filtration, and recrystallized from (EtOH). **10** was obtained as red crystal, yield (67%); m.p. > 320–322 °C; IR (ν_max_): 1737 (C=O), 2206 (CN), 3390 (C=N), cm^−1;^ ^1^H-NMR (400 MHz, DMSO-*d*_6_) δ (ppm): 1.91 (s, 3H, CH_3_), 6.70 (d, 1H, *J* = 8.6, CH), 7.42–7.90 (m, 5H, Ar-H), 8.13 (d, 1H, *J* = 8.6, CH), 11.97 (s, 1H, NH); ^13^C-NMR (100 MHz, DMSO-*d*_6_) δ (ppm): 14.46, 106.18, 98.86, 115.20, 129.86, 151.36, 162.89. MS *m/z* (%): 466 [M^+^, 45%]; Anal. Calcd for C_25_H_14_N_4_O_2_S_2_: C, 64.36; H, 3.02; N, 12.01; S, 13.75; Found: C, 64.38; H, 3.02; N, 12.03; S, 13.75.

### 6,6′-(3-methyl-4-phenylthieno[2,3-b]thiophene-2,5-dicarbonyl)bis(3-oxo-2,3-dihydropyridazine-4-carboxylic acid) (**9**)

Procedure A: To a solution of compound 8 (0.5 mmol, 0.278 g) in a mixture of dioxane (15 mL) and HCl (2 mL). A solution of NaNO_2_ (0.069 g, 1 mmol) and sodium acetate (1.5 mmol) in water (6 mL) was added dropwise. The resulting reaction mixture was heated under reflux for 4 h. The solid product was collected by filtration, and recrystallized from dioxane.

Procedure B: Coupling reaction to a stirred solution of the compound 8 (0.5 mmol, 0.28 g) in dioxane (15 mL) and the mixture was cooled in an ice bath to 0–5 °C. A solution of the benzenediazonium chloride, [freshly prepared by diazotizing the respective aniline (1 mmol) in hydrochloric acid (0.28 mL) with sodium nitrite solution (2 mmol) in (4 mL) water] was added drop wise over 20 min. The whole mixture was then left in a refrigerator overnight. The solid product was collected by filtration, and recrystallized from (EtOH). 9 was obtained as brown crystal, yield (72^a^, 68^b^%); m.p. > 320–322 °C; IR (ν_max_): 1579 (C=N),1666–1643 (C=O), 3444 (OH), 3444 (NH) cm^−1;^ ^1^H-NMR (400 MHz, DMSO-*d*_6_) δ (ppm): 2.08 (s, 3H, CH_3_), 6.68 (s, 1H, NH) 7.42–7.80 (m, 5H, Ar-H), 8.09 (s, 1H, CH), 8.35 (s, 1H, OH); ^13^C-NMR (100 MHz, DMSO-*d*_6_) δ (ppm): 14.89, 125.68, 128.92, 129.96, 131.56, 149.85, 152.16, 163.76, 178.22, 191.62; MS *m/z* (%): 562 [M^+^, 45%]; Anal. Calcd for C_25_H_14_N_4_O_8_S_2_: C, 53.38; H, 2.51; N, 9.96; S, 11.40; Found: C, 53.40; H, 2.50; N, 9.94; S, 11.42.

## 4. Conclusions

In conclusion, the present investigation describes an efficient method for access toward novel bis-(heterocycles) containing two biologically active moieties. We believe that these new series of bis-(hetrocycles) may exhibit potentially diverse useful applications in the field of medicinal chemistry. Also, development of the above synthetic methodology should lead to the synthesis of a large number of bis-(hetrocycles) with a wide variety of substituent as well as different bridges.
